# LncRNA MIR4435-2HG targets desmoplakin and promotes growth and metastasis of gastric cancer by activating Wnt/β-catenin signaling

**DOI:** 10.18632/aging.102164

**Published:** 2019-09-04

**Authors:** Haiyong Wang, Mengjie Wu, Yimin Lu, Kuifeng He, Xiaolu Cai, Xiongfei Yu, Jun Lu, Lisong Teng

**Affiliations:** 1 Department of Surgical Oncology, First Affiliated Hospital, Zhejiang University, Zhejiang, China; 2 Key Laboratory of Precision Diagnosis and Treatment for Hepatobiliary and Pancreatic Tumor of Zhejiang Province, First Affiliated Hospital, Zhejiang University, Zhejiang, China

**Keywords:** gastric cancer, MIR4435-2HG, β-catenin, desmoplakin, lncRNA

## Abstract

Long non-coding RNAs (lncRNAs) have been implicated in the pathogenesis of gastric cancer; however, their mechanisms of action remain largely unknown. The aim of this study was to identify lncRNAs involved in the tumorigenesis of gastric cancer and to investigate the signaling pathways they affect. Using microarray and RT-qPCR analyses, candidate lncRNAs were screened in paired gastric cancer tissues. The analysis revealed MIR4435-2HG to be markedly up-regulated in gastric cancer samples compared to normal stomach specimens. Increased MIR4435-2HG expression was associated with aggressive clinicopathologic features and unfavorable tumor stage. Functional experiments showed that MIR4435-2HG up-regulation enhanced gastric cancer cell proliferation, clonogenicity, and migration and invasion *in vitro*, as well as tumorigenicity in mice. Using RNA pull-down and mass-spectrometry analyses we found and verified a direct and novel interaction between MIR4435-2HG and desmoplakin (DSP), the most abundant desmosomal protein. Overexpression and knockdown experiments revealed opposing roles for DSP and MIR4435-2HG, unmasking a cascade through which MIR4435-2HG binds to and inhibits DSP, leading to activation of WNT/β-catenin signaling and epithelial-mesenchymal transition in gastric cancer cells. We propose that the MIR4435-2HG/DSP/WNT axis serves as a critical effector of carcinogenesis and progression of gastric cancer, and could be exploited therapeutically to improve patients’ outcomes.

## INTRODUCTION

Gastric cancer (GC) is one of the most aggressive human malignancies, representing the fourth most common cancer and the second leading cause of cancer-related mortality worldwide [1]. Although the diagnosis and therapy of GC have improved, progressive GC remains highly lethal because of its aggressive metastatic behavior and the fact that it is often diagnosed at an advanced stage [2, 3]. Therefore, understanding the molecular mechanisms underlying GC and discovering specific biomarkers to monitor its progression are of great clinical significance.

Long noncoding RNAs (lncRNAs) are transcribed RNA molecules longer than 200 nucleotides in length [4] that act as scaffolds to affect chromatin assembly and remodeling, gene transcription, and protein translation or stability [5, 6]. Multiple lines of evidence suggest lncRNAs have important roles in tumor biology, typically leading to abnormal expression of gene products involved in the progression of human cancers [7]. For instance, dysregulation of HOTAIR, one of the best studied lncRNAs, can lead to altered target gene expression and contribute to the invasiveness and metastasis of different cancers [8, 9]. Moreover, some lncRNAs can be specific tumor biomarkers. One example is PCA3, which has been approved by the FDA as a diagnostic biomarker in prostate cancer [10].

The lncRNA MIR4435-2HG (also known as AK001796 and LINC00978) is encoded on human chromosome 2q13. Acting as an oncogene, MIR4435-2HG was initially characterized by its involvement in resveratrol-mediated cell growth inhibition in lung cancer [11]. Subsequently, MIR4435-2HG expression was found to positively correlate with poor outcome in breast cancer patients [12]. Recent studies, on the other hand, reported that MIR4435-2HG promoted GC growth and suggested its relevance as a diagnostic marker [13, 14]. Nevertheless, the molecular mechanisms linking MIR4435-2HG and GC remain largely undefined.

Using lncRNA expression microarray screening, RNA pull-down assays, and functional *in vitro* and *in vivo* studies, we characterized a novel interaction between MIR4435-2HG and desmoplakin (DSP), a desmosome-associated protein, in human GC. Knockdown and overexpression experiments confirmed that increased MIR4435-2HG expression in GC inhibits DSP activity, promoting Wnt/β-catenin signaling and epithelial-mesenchymal transition (EMT). Thus, our study provides new insight into the molecular mechanisms by which MIR4435-2HG contributes to the pathogenesis of GC.

## RESULTS

### Up-regulation of MIR4435-2HG in GC is correlated with advanced tumor stage

**Figure 1:**
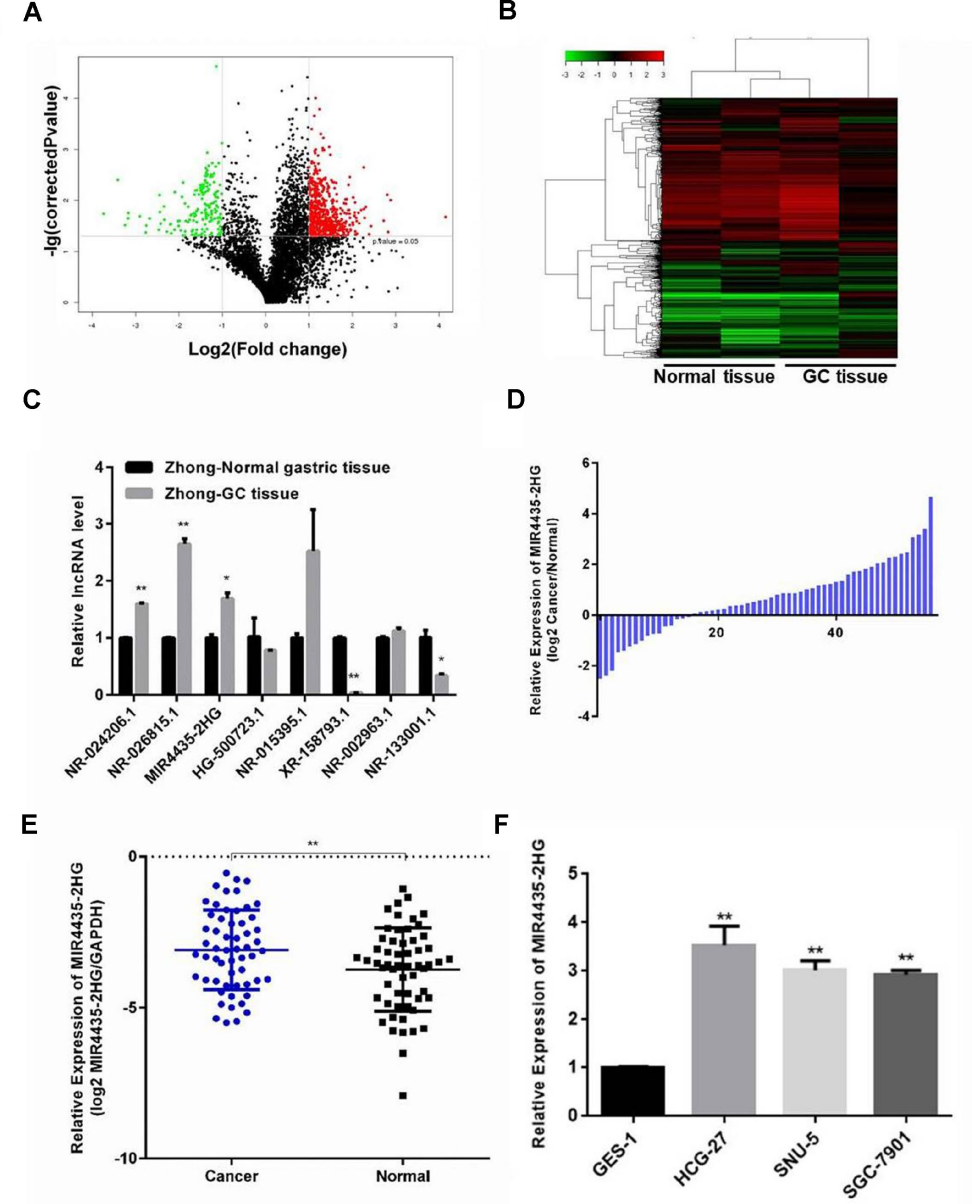
**Expression of MIR4435 is up-regulated in GC tissues and cell lines.** (**A**) Volcano plot of lncRNAs illustrating the difference in lncRNA expression between GC tissues and paired normal gastric tissues. The red and green symbols depict significantly up- and down-regulated lncRNAs in GC (*P* < 0.05). (**B**) Heat map and hierarchical clustering analysis showing the different lncRNA expression profiles in GC and normal samples. The relative expression from high to low level is shown with red and green color. (**C**, **D**) Validation of the differential expression of 8 lncRNAs in Zhong cancer tissue and paired normal gastric tissue using RT-qPCR (*P < 0.05, **P < 0.01). (**E**) Detection of MIR4435-2HG expression in 57 GC samples and 57 adjacent normal gastric samples using RT-qPCR. MIR4435-2HG levels were normalized to GAPDH and expressed in terms of the threshold cycle (CT) ratio (**P < 0.01). (**F**) Basal expression of MIR4435-2HG in GES-1, HGC-27, SNU-5, and SGC-7901 cells.

To identify lncRNAs potentially involved in GC progression, lncRNA expression microarray analysis was performed on four human GC samples and paired normal gastric tissues. The analysis revealed several up-regulated and down-regulated lncRNAs (Figure 1A). In particular, five significantly up-regulated lncRNAs, p37203_v4 (NR_024206.1), p33536 (NR_026815.1), p34737_v4 (NR_024373.1, also known as MIR4435-2HG), p15510 (HG500723.1), and p9290 (NR_015395.1), and three significantly down-regulated lncRNAs, p30133 (XR_158793.1), p37592_v4 (NR_002963.1), and p34460_v4 (NR_133001.1) were detected in GC samples compared to their respective non-cancerous counterparts (P < 0.05; Figure 1B). The expression of the above lncRNAs was validated in all samples by RT-qPCR (Figure 1C and Supplementary Figure 1A). Furthermore, we found that MIR4435-2HG expression was considerably up-regulated in 57 additional GC specimens, compared to matched samples of normal gastric mucosa (Figure 1D and 1E). To assess the clinical significance of MIR4435-2HG, a high-expression group (n = 30) and a low-expression group (n = 27) were defined based on median MIR4435-2HG expression levels. Correlation analysis revealed that MIR4435-2HG expression was positively associated with TNM stage (Table 1).

**Table 1:** Clinicopathologic characteristics of MIR4435-2HG expression in GC patients.

	**No. patients**	**Low expression (n = 27)**	**High expression (n = 30)**	***P* value**
Gender				
Male	42	22	20	0.2047^a^
Female	15	5	10	
Age				
≤60	18	7	11	0.3837^a^
>60	39	20	19	
T stage				
T1-2	10	9	1	0.0087^b^
T3-4	47	18	29	
N stage				
N0-1	19	12	7	0.0914^a^
N2-3	38	15	23	
M stage				
M0	50	22	28	0.3385^b^
M1	7	5	2	
Tumor size			
≤5cm	33	16	17	0.8431^a^
>5cm	24	11	13	
Differentiation			
Well to moderate	11	7	3	0.2412^b^
Poor	46	20	26	
AJCC stage				
I-II	13	10	3	0.0346^b^
III-IV	44	17	27	

a: chi-square test; b: Yates’ continuity corrected chi-square test.

In addition, MIR4435-2HG expression was examined in GC cell lines and normal gastric epithelial cells by RT-qPCR. Compared to normal gastric epithelial GES-1 cells, up-regulation of MIR4435-2HG was observed in the HGC-27, SNU-5, and SGC7901 GC cell lines (Figure 1F). These data suggested that up-regulation of MIR4435-2HG might contribute to GC pathogenesis.

### MIR4435-2HG knockdown inhibits growth and invasion of GC cells *in vitro*

**Figure 2:**
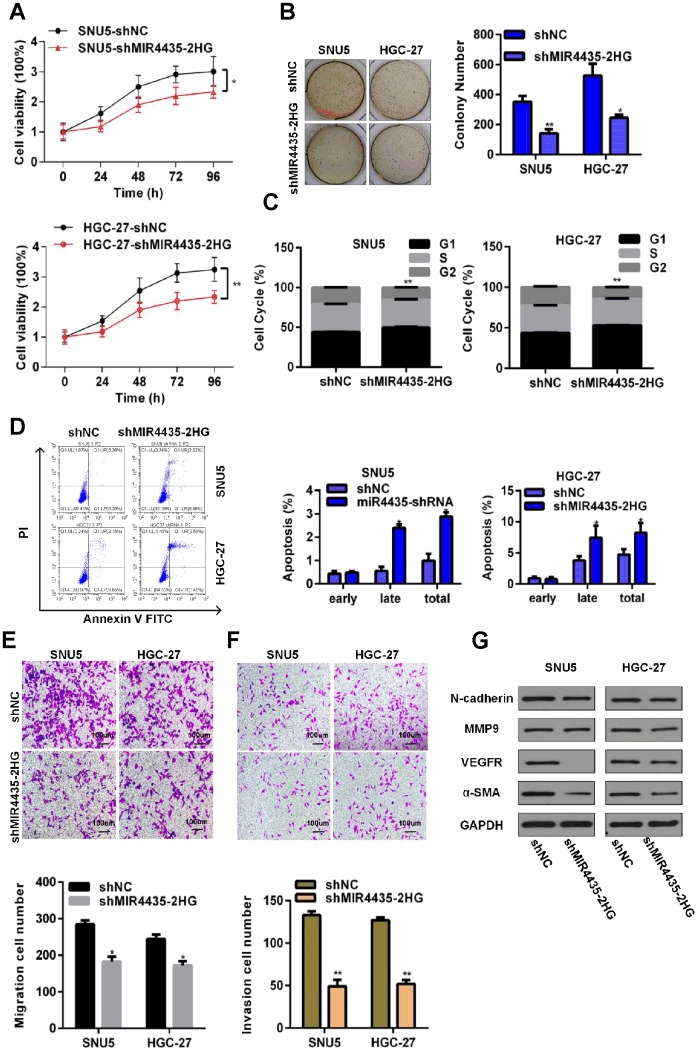
**Depletion of MIR4435-2HG in GC cells inhibits cell growth and metastasis *in vitro*.** (**A**) MIR4435-2HG knockdown inhibited cell proliferation (CCK8 assays) (*P < 0.05, **P < 0.01). (**B**) Colony formation assays were used to evaluate SNU5 and HGC-27 cell proliferation after inhibiting expression of MIR4435-2HG (*P < 0.05, **P < 0.01). (**C**) The cell cycle was analyzed using flow cytometry after knocking down MIR4435-2HG (**P < 0.01). (**D**) Flow cytometric apoptosis assays were used to analyze the incidence of apoptosis after silencing MIR4435-2HG (*P < 0.05). (**E**) MIR4435-2HG inhibition decreased cell migration in transwell assays (*P < 0.05). (**F**) Transwell assays used to assess the invasiveness of cells with downregulated MIR4435-2HG (**P < 0.01). (**G**) Expression of N-cadherin, MMP-9, VEGF and α-SMA in SNU5 and HGC-27 cells was detected by WB after decreasing MIR4435-2HG expression.

To investigate the role of MIR4435-2HG in GC, its expression was stably knocked down in two GC cell lines (SNU5 and HGC-27) by transfection with MIR4435-2HG-specific shRNA (shMIR4435-2HG). Successful knockdown was confirmed by RT-qPCR (Supplementary Figure 1B). Compared to negative controls (NC, i.e. cells transfected with scrambled shRNA), both SNU5-shMIR4435-2HG cells and HGC-27-shMIR4435-2HG cells showed lower proliferation rates in CCK-8 assays (Figure 2A). Likewise, colony formation capacity was also decreased in both cell lines after transfection with MIR4435-2HG shRNA (Figure 2B). Meanwhile, flow cytometry analyses showed a higher percentage of cells in G1 phase and increased apoptosis rates after MIR4435-2HG-knockdown (Figure 2C and 2D). These findings indicate that down-regulation of MIR4435-2HG can arrest cells in G1 phase and promote apoptosis, effectively decreasing proliferation.

To evaluate whether down-regulation of MIR4435-2HG would affect the migration and invasion of SNU5 and HGC-27 cells, we conducted transwell assays. Results showed that MIR4435-2HG suppression attenuated migration and invasion in both cell lines (Figure 2E and 2F). To investigate the molecular bases of this inhibition, the expression of EMT-related proteins was examined by western blotting (WB). Markedly decreased expression of N-cadherin, VEGF, and α-SMA was observed in sh-MIR4435-2HG-transfected SNU5 (Figure 2G). Meanwhile, significantly decreased expression of MMP9, VEGF, and α-SMA was observed in sh-MIR4435-2HG-transfected HGC27 (Figure 2G). These findings suggest that MIR4435-2HG promotes migration and invasion of GC cells by inducing EMT.

### MIR4435-2HG promotes GC cell tumorigenesis *in vivo*

**Figure 3:**
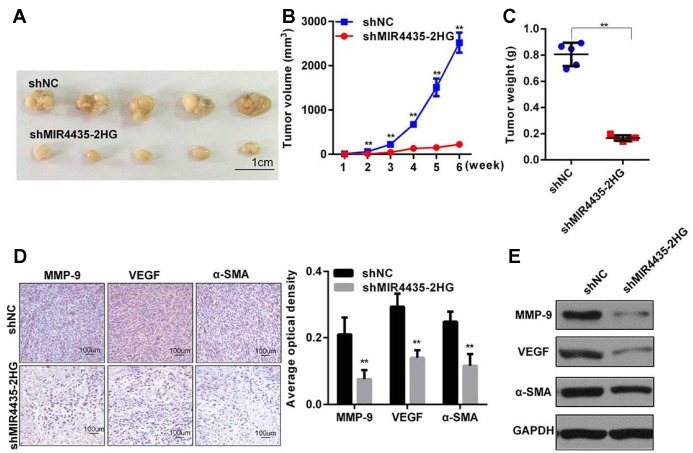
**MIR4435-2HG promotes gastric cancer cell tumorigenesis *in vivo*.** (**A**) MIR4435-2HG knockdown inhibited subcutaneous tumor formation in nude mouse models. Compared to the HGC-27/shNC group, the HGC-27/shMIR4435-2HG group showed smaller tumor sizes (*n=5*). (**B**) Quantitative comparison was made between the shNC and shMIR4435-2HG groups at weekly intervals using t-tests. Data are means ± SD for five samples (**P < 0.01). (**C**) Individual tumor weights from mice in the two groups (**P < 0.01). (**D**) Expression of N-Cadherin, MMP-9, VEGF and α-SMA in the two groups was detected using IHC. (**E**) Expression of MMP-9, VEGF and α-SMA in two groups were detected by WB and quantitated (**P < 0.01).

To examine the effect of MIR4435-2HG on GC proliferation *in vivo*, HGC-27 cells stably transfected with sh-MIR4435 or scrambled control vectors (verified by RT-qPCR; Supplementary Figure 1C) were inoculated into nude mice. Tumor volumes were measured every week after injection. By week 6, tumor weights and volumes were significantly smaller in mice injected with HGC-27/sh-MIR4435-2HG cells (Figure 3A–3C).

Furthermore, several proteins important for cancerous progression were detected in xenografted tumors by IHC and WB assays. Results showed that the expression of E-Cadherin, MMP-9, VEGF, and α-SMA in HGC-27/sh-MIR4435-2HG tumors was significantly lower than in the HGC-27/shNC group (Figure 3D and 3E). These results, which are in accord with our *in vitro* findings, strongly suggest that MIR4435-2HG contributes to the progression of GC *in vivo*.

### MIR4435-2HG binds to DSP and inhibits its expression

**Figure 4:**
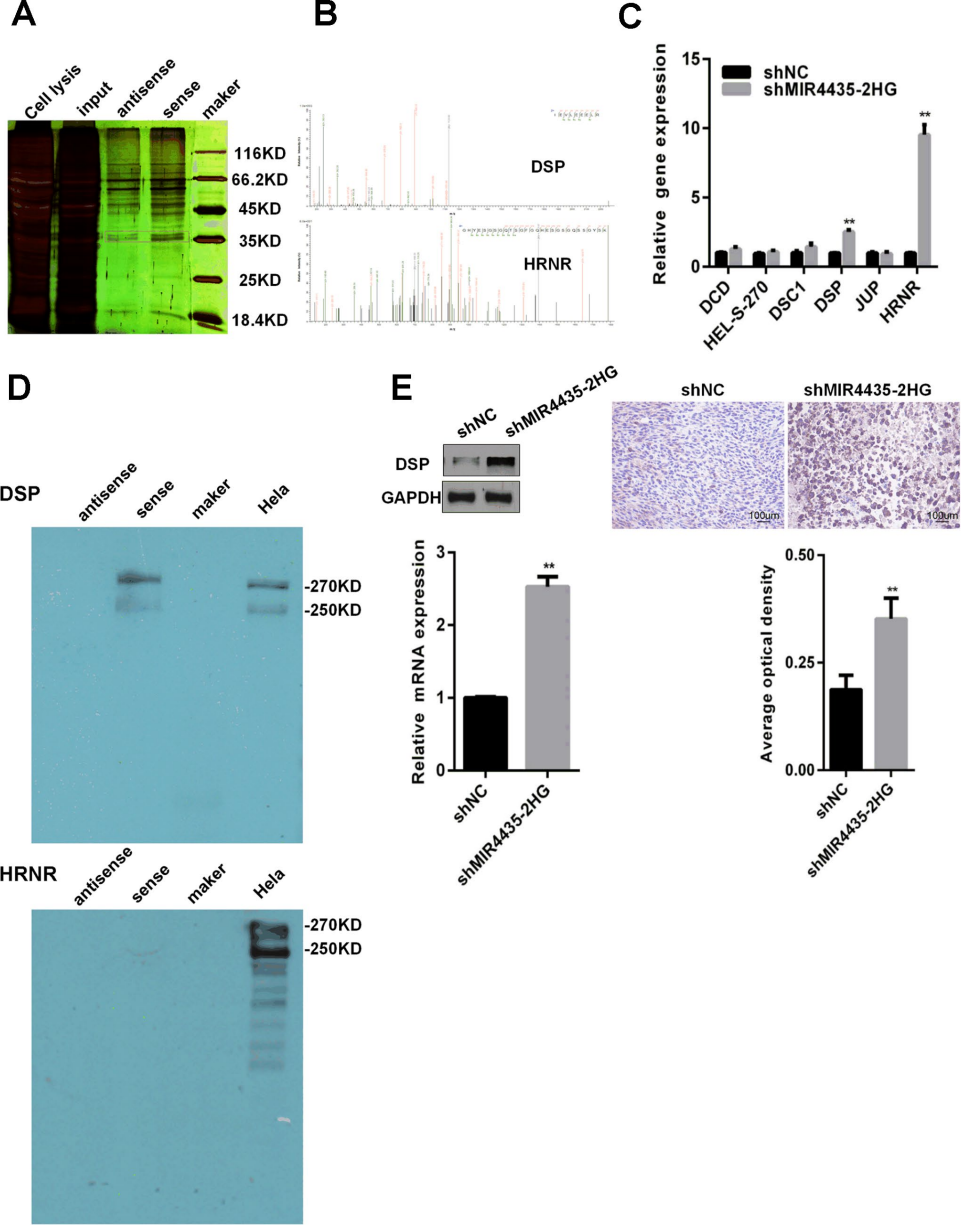
**MIR4435-2HG binds to DSP protein and reduces DSP expression.** (**A**) RNA pull-down experiments were performed to identify proteins interacting with MIR4435-2HG. Silver staining revealed a specific band associated with biotinylated sense MIR4435-2HG. (**B**) The biotinylated sense MIR4435-2HG band was excised, digested, and subjected to mass spectrometry, which identified DSP and HRNR as the MIR4435-2HG-interacting proteins. (**C**) Levels of DSP and HRNR were confirmed in the shMIR4435-2HG (HGC-27 cells) and control groups using RT-qPCR (**P < 0.01). (**D**) The interaction of DSP with MIR4435-2HG or HRNR with MIR4435-2HG by RNA pull-down assay. DSP was detected within sense MIR4435-2HG pull-down protein complexes but not with antisense MIR4435-2HG. (**E**) Expression levels of DSP within HGC-27/shMIR4435-2HG-xenografted and HGC-27/shNC-xenografted tumors were detected using WB and IHC (**P < 0.01).

Research showed that lncRNAs may regulate intracellular signaling through their interaction with RNA-binding proteins [15, 16]. Therefore, RNA-pull-down experiments were performed to search for MIR4435-2HG-interacting proteins. A specific band associated with biotinylated sense MIR4435-2HG was identified by silver staining after the pull-down assay (Figure 4A, red box). This band was cut out, digested, and subjected to mass spectrometry, which identified DSP, DCD, DSC1, HEL-S-270, HRNR, and JUP as MIR4435-2HG-interacting proteins (Figure 4B, Supplementary Figure 1D). Next, we examined the effect of MIR4435-2HG silencing on the expression of the above proteins by RT-qPCR. Results showed that DSP and HRNR were up-regulated in HGC-27 cells transfected with shMIR4435-2HG (Figure 4C). Furthermore, we verified the interaction of DSP with MIR4435-2HG or HRNR with MIR4435-2HG by RNA pull-down assay; DSP was detected in sense, but not antisense, MIR4435-2HG pull-down protein complexes (Figure 4D). In contrast, HRNR could not interact with MIR4435-2HG. Finally, we studied the interaction between MIR4435-2HG and DSP in HGC-27/shMIR4435-2HG and HGC-27/shNC xenografts by WB and IHC assays. Results demonstrated that the expression of DSP was inversely related to that of MIR4435-2HG (Figure 4E). These data suggest that MIR4435-2HG interacts with DSP, reducing its expression.

### DSP inhibits GC proliferation and metastasis through Wnt/β-catenin signaling inhibition

**Figure 5:**
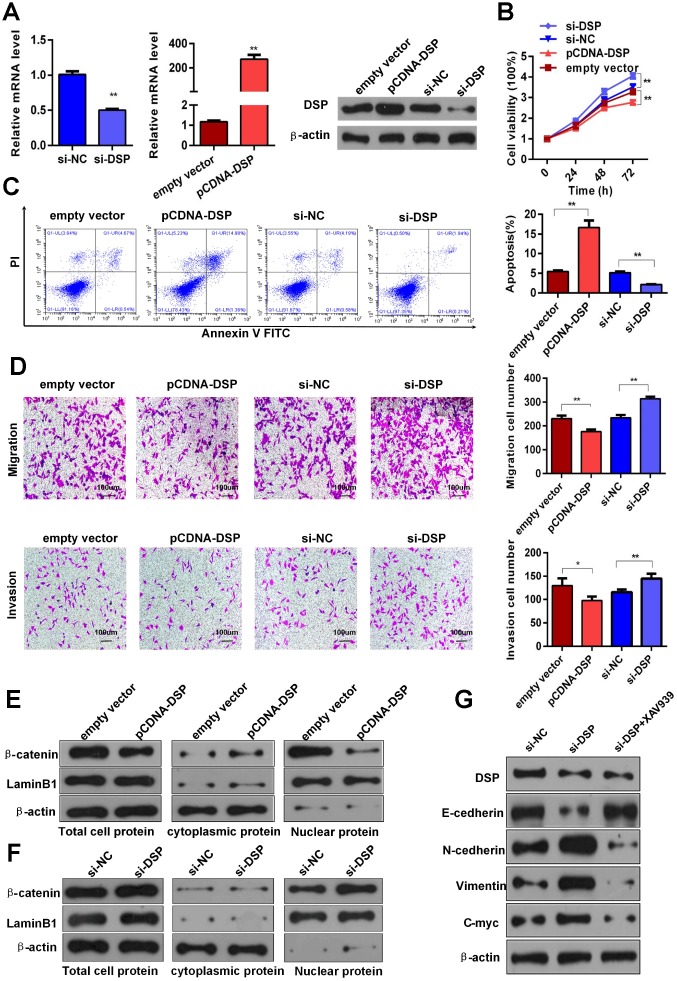
**DSP inhibits GC growth and metastasis via Wnt/β-catenin signaling.** (**A**) Depletion and overexpression of DSP were verified using RT-qPCR and WB. (**B**) CCK8 assays were performed after knockdown and overexpression of DSP (**P < 0.01). (**C**) Flow cytometric apoptosis assays were used to assess cell apoptosis after silencing and overexpression of DSP (**P < 0.01). (**D**) Transwell assays were used to determine the invasiveness of cells with down-regulated and up-regulated DSP (*P < 0.05, **P < 0.01). (**E**–**F**) Total, cytoplasmic and nuclear levels of β-catenin and lamin B1 were detected by WB after promoting or inhibiting DSP expression. (**G**) Expression levels of E-cadherin, N-cadherin, vimentin and c-myc were determined using WB after DSP knockdown or combined treatment with DSP siRNA and a Wnt/β-catenin inhibitor (XAV939a).

To clarify the effects of DSP on GC, we performed knockdown and overexpression assays in HGC-27 cells using siRNA (si-DSP/si-NC) or pCDNA3.1-DSP vector transfection (pCDNA-DSP/empty vector), respectively (Figure 5A). CCK8 assays showed that DSP knockdown and overexpression promoted and inhibited, respectively, the proliferation of HGC-27 cells (Figure 5B). Meanwhile, flow cytometry revealed that DSP down-regulation led to lower apoptotic rates, while DSP up-regulation promoted apoptosis of GC cells (Figure 5C). In transwell assays, DSP knockdown enhanced migration and invasion of HGC-27 cells, whereas DSP overexpression had the opposite effect (Figure 5D). These findings suggest that DSP expression restricts growth and metastasis of GC cells.

A previous study reported that DSP could function as a tumor suppressor through inhibition of the Wnt/β-catenin pathway in human lung cancer [17]. Considering the important role of Wnt/β-catenin pathway dysregulation in the progression of various tumors, including GC [18, 19], we next examined whether DSP could inhibit GC growth and metastasis by interfering with Wnt/β-catenin signaling. WB analyses revealed that total and nuclear expression of β-catenin was reduced in HGC-27 cells overexpressing DSP, while the expression of this protein in the cytoplasm was instead increased (Figure 5E). Contrast to these results, DSP knockdown increased both total and nuclear β-catenin levels (Figure 5F). Furthermore, we found evidence that DSP could inhibit EMT signaling. As shown by WB, DSP knockdown increased the expression of N-cadherin, vimentin, and c-Myc, and decreased E-cadherin expression. Accordingly, changes in N-cadherin, vimentin, and c-Myc were attenuated, and E-cadherin expression was rescued, after treatment with si-DSP and XAV939 (a Wnt/β-catenin attenuated, and E-cadherin expression was rescued, after treatment with si-DSP and XAV939 (a Wnt/β-catenin inhibitor) (Figure 5G). Meanwhile, the expressions of c-myc and β-catenin were obviously upregulated, whereas the level of DSP was obviously downregulated in GC specimens, compared with paired normal gastric tissues (Supplementary Figure 2A and 2B). Together, these data indicate that the capacity of DSP to inhibit GC growth and metastasis is in large part attributable to its ability to suppress Wnt/β-catenin signaling.

### MIR4435-2HG regulates the Wnt/β-catenin signaling pathway through DSP to promote GC tumorigenesis

**Figure 6:**
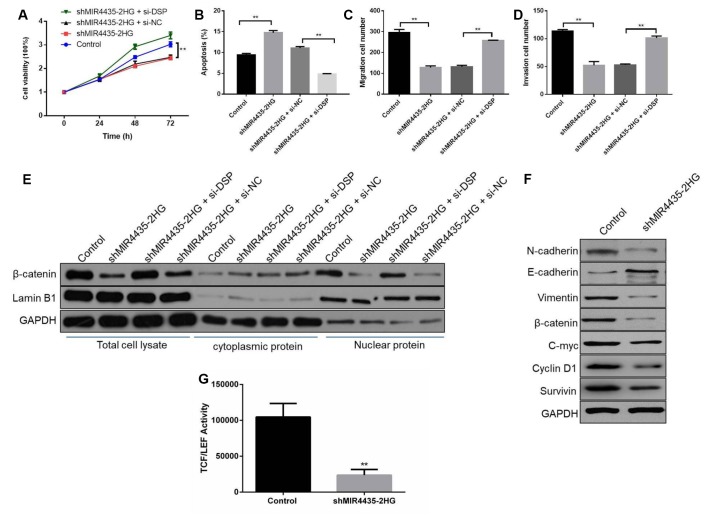
**MIR4435-2HG regulates Wnt/β-catenin signaling through DSP to promote GC tumorigenesis and progression.** (**A**) CCK8 assays were used with the HGC-27 cells co-transfected with shMIR4435-2HG and si-DSP (**P < 0.01). (**B**) The incidence of apoptosis among HGC-27 cells co-transfected with shMIR44352HG and si-DSP was determined using flow cytometry (**P < 0.01). (**C**, **D**) Transwell assays were used to assess cell migration and invasion in the group co-transfected with shMIR4435-2HG and si-DSP (**P < 0.01). (**E**) WB was performed to assess expression of β-catenin and lamin B1. (**F**) Levels of E-cadherin, N-cadherin, vimentin, c-Myc, cyclin D1, survivin and β-catenin within tumor tissue were determined using WB. (**G**) TCF/LEF transcriptional activity was determined by dual-luciferase assays in HGC-27 cells in response to MIR4435-2HG inhibition.

To test the hypothesis that MIR4435-2HG regulates the Wnt/β-catenin signaling through DSP to promote GC tumorigenesis and progression, we conducted rescue assays by co-transfecting shMIR4435-2HG and si-DSP into HGC-27 cells. As expected, CCK8 assays showed that DSP silencing partly rescued growth inhibition induced by shMIR4435-2HG (Figure 6A). In turn, apoptosis was reduced in cells co-transfected with shMIR44352HG and si-DSP, compared to cells transfected with shMIR4435-2HG or scrambled NC shRNA alone (Figure 6B and Supplementary Figure 1E). Similarly, partial restoration of migratory and invasive capacity was seen in MIR4435-2HG knockdown cells after si-DSP transfection. Under this condition, the number of migrating and invading cells was also lower in co-transfected with shMIR44352HG and si-DSP group than that transfected with shMIR4435-2HG (Figure 6C, 6D and Supplementary Figure 2C).

On the other hand, the expression of β-catenin was up-regulated in HGC-27 cells co-transfected with shMIR4435-2HG and si-DSP, compared with cells transfected with shMIR4435-2HG alone (Figure 6E). Moreover, knockdown of MIR4435-2HG increased E-cadherin and decreased N-cadherin, vimentin, c-Myc, β-catenin, cyclin D1 and survivin expression in tumor xenografts (Figure 6F and Supplementary Figure 2D). Meanwhile, the luciferase reporter assay revealed that inhibition of MIR4435-2HG markedly decreased the transactivating activity of β-catenin in HGC-27 cells (Figure 6G). In summary, these results indicate that MIR4435-2HG promotes GC growth and metastasis by activating Wnt/β-catenin signaling via DSP targeting.

## DISCUSSION

It is now widely established that mammalian genomes produce thousands of lncRNAs, some of which may contribute to carcinogenesis and metastasis [18, 20–22]. However, the normal functions of lncRNAs and the basis of their abnormal expression in tumors remain obscure. Through lncRNA expression microarray screening of clinical GC specimens, followed by expression and functional analyses *in vitro* and *in vivo*, our study identified the lncRNA MIR4435-2HG as a putative mediator of GC.

Up-regulation of MIR4435-2HG was observed in both human GC samples and GC cell lines, and was associated with more aggressive tumor invasion and late TNM stage. Accordingly, increased MIR4435-2HG expression was found to promote proliferation and restrict apoptosis of GC cells *in vitro*, and to stimulate tumorigenesis in mice. These results suggest the oncogenic potential of MIR4435-2HG on GC, consistent with a similar role reported for lung cancer, breast cancer, and esophageal squamous cell carcinoma [12, 23, 24]. Thus, MIR4435-2HG joins the list of several lncRNAs, including H19, HOTAIR, and GAPLINC, reported to influence GC development, progression, and metastasis [25–27].

One of the MIR4435-2HG-interacting proteins in our RNA pull-down assays, also verified by WB, was DSP, a founding member of the plakin family of proteins and an important component of desmosomal plaques [28]. Several studies suggested that loss of DSP is not uncommon in human tumors and may be an early step in carcinogenesis [29–32]. DSP has been reported to act as a tumor suppressor by inhibiting the Wnt/β-catenin signaling pathway in human lung cancer [17]. While dysregulated Wnt/β-catenin signaling is commonly observed in GC [18, 19, 33] a role for DSP in this malignancy has not been reported so far. Based on these data and our current findings, we speculated that MIR4435-2HG targets DSP to affect Wnt/β-catenin signaling and promote GC. Indeed, our results showed that MIR4435-2HG expression was closely associated with that of β-catenin and lamin B1, two key players in the activation of the WNT canonical pathway [34]. After ligation of WNT proteins to their respective cell surface receptors, β-catenin is released from the degradation complex and translocated into the cell nucleus to effect gene expression changes [35]. Our study indicated that up-regulation of DSP in GC cells decreased the expression of nuclear β-catenin, thus suppressing the activation of the Wnt/β-catenin pathway. Moreover, DSP knockdown led to decreased expression of N-cadherin, vimentin, and c-Myc, and increased expression of E-cadherin, suggesting that DSP inhibits the metastatic potential of GC cells by preventing EMT. Accordingly, we found that the EMT promoted by DSP knockdown was attenuated by a Wnt/β-catenin inhibitor. Altogether, these results revealed that up-regulation of the lncRNA MIR4435-2HG in GC cells reduces DSP activity, and this event stimulates the growth and metastasis of GC by inducing Wnt/β-catenin signaling and EMT. To our knowledge, this is the first study that reports the interaction between MIR4435-2HG and DSP, exposing a novel mechanism at play in the progression and metastasis of GC and perhaps other tumors.

Further studies are needed to validate MIR4435-2HG as a potential diagnostic or prognostic maker for GC, as well as its relevance as a therapeutic target.

## MATERIALS AND METHODS

### Tissue samples and cell lines

GC specimens were obtained from patients of the First Affiliated Hospital of Zhejiang University, with confirmatory diagnoses based on histopathology. Informed consents prior to surgery were obtained for every patient. Human gastric cell lines (SNU5, HGC27 and SGC7901) and a normal gastric epithelial cell line (GES-1) were purchased from Shanghai Cell Bank of Chinese Academy of Sciences (Shanghai, China). HGC27, SGC7901, and GES-1 cells were cultured in RPMI 1640; SNU5 cells were cultured in IMDM medium with 10% fetal bovine serum (Gibco).

### LncRNA expression microarray

The Affymetrix Human Gene 2.0 ST Array (Affymetrix, Santa Clara, CA, USA) was used to analyze lncRNA in four paired GC and normal gastric samples. Sample preparation and procedures were preformed based on the manufacturer’s standard protocols. Raw data was extracted by Feature Extraction software 10.7 (Agilent Technologies, Inc.) and normalized by Quantile algorithm using the GeneSpring GX v11.5 software package (Agilent Technologies). Genes with a fold change >2.0 or <0.5 (P < 0.05) were noted as statistically significant.

### RNA extraction and quantitative reverse transcription PCR (RT-qPCR)

Procedures for RT-qPCR are described in Supplementary Methods. All primer sequences used in this study are shown in Supplementary Table 1.

### Cell transfections

Plasmid vectors (pCDNA-DSP, sh-MIR4435-2HG, and empty vectors) and siRNA (si-DSP and scrambled negative control siRNA) were transfected into SNU5 or HGC-27 cells using Lipofectamine 2000 (Invitrogen, Carlsbad, California, USA) in Opti-MEM medium (Gibco, Carlsbad, California, USA) according to manufacturer’s instructions. The siRNA targeting DSP was synthesized by Invitrogen. All siRNA and shRNA sequences are shown in Supplementary Table 2.

### Cell proliferation, flow cytometry, and transwell assay

Procedures for these assays are described in Supplementary Methods.

### *In vivo* tumorigenesis

Ten male athymic BALB/c mice aged 3-4 weeks were purchased from Hubei Provincial Center for Disease Control and Prevention. All animal experiments were performed according to the protocols approved by the Institutional Animal Care and Use Committee at the Zhejiang University. For xenograft models, 4 × 10^6^ HGC-27 cells transfected with sh-MIR4435 vector or sh-NC were injected subcutaneously in the right flank of mice (five mice per group). Tumor size was measured every 7 days using the equation V(mm^3^) = 0.5 × a × b^2^ (V, volume; a, length; b, width). After 6 weeks, the mice were sacrificed and tumors were weighed and processed for qPCR, immunohistochemistry, or western blot analyses.

### Immunohistochemical (IHC) assay

Description of the IHC assay is provided in Supplementary Methods.

### Western blot (WB) analyses

WB techniques are described in Supplementary Methods.

### RNA pull-down assay and mass spectrometry analysis

The DNA templates of sense and antisense MIR4435 fragments used for transcription were cloned using PCR; primer sequences are listed in Supplementary Table 1. Sense and antisense MIR4435 strands were *in vitro* transcribed with the TranscriptAid T7 High Yield transcription Kit (Thermo Fisher Scientific, USA) and then labeled with desthiobiotin using Pierce RNA3′ End Desthiobiotinylation Kit (Thermo Fisher Scientific, USA). RNA pull-down was performed using the Pierce™ Magnetic RNA-Protein Pull-Down Kit (Thermo Fisher Scientific, USA) and protein bands of interest were analyzed with an UltiMate 3000 Rapid Separation LC (RSLC) system.

### Luciferase reporter assay

HGC-27 cells were plated into 24-well plates and incubated for 24 h. The cells were co-transfected with wild-type TCF reporter plasmid TOP Flash (Upstate Biotechnology, Lake Placid, NY, USA) or mutated TCF reporter plasmid FOP flash (Upstate Biotechnology), and shMIR4435-2HG plasmid or NC using Lipofectamine 2000 (Invitrogen, Carlsbad, CA, USA) respectively. After 48 h of incubation, the relative luciferase activities in cell lysates were detected using a Dual-Luciferase Reporter Assay System (Promega, Madison, WI, USA).

### Statistical analyses

All experiments were separately repeated 3 times. All data are expressed as the mean ± SD. Each bar represents the mean ± SD of 3 independent experiments. Statistical significance between two or multiple groups was analyzed by t-test or one-way ANOVA using GraphPad Prism 6.0. Statistical significance was assumed if *P* < 0.05.

## Supplementary Material

Supplementary Material

Supplementary Figures

Supplementary Tables
