# Supplementary material for: LncRNA MIR4435-2HG targets desmoplakin and promotes growth and metastasis of gastric cancer by activating Wnt/β-catenin signaling
Source: Aging (Albany NY). 2019 Sep 4;11(17):6657–73. doi: 10.18632/aging.102164 (PMC6756883; doi:10.18632/aging.102164)
Supplement: Supplementary Material [file aging-11-102164-s002.pdf]

SUPPLEMENTARY METHODS

RNA extraction and quantitative real-time PCR(RT-qPCR)

Total RNA was extracted from tissues or cells with TRIzol reagent (Invitrogen, Carlsbad, California, USA) and then reverse transcribed to cDNA using the RevertAid First Strand cDNA Synthesis Kit (Thermo Fisher Scientific, USA). Flourescein qPCR Master Mix (Thermo Fisher Scientific, USA) was used for RT-qPCR. The GAPDH was used as internal control.

Cell proliferation (cck8, and soft agar colony formation assay)

Cell viability and proliferation ability was assessed using CCK-8 assay, or clone formation assay. For CCK-8 assay, cells were seeded in 96-well plates. After the cell growth, the culture medium was removed and 10 μl CCK8 and 100 μl culture medium were added to each well, followed by an incubation of 3 hours. The absorbance was measured at 450nm; for soft agar colony formation assay, GC cells were seeded in 6-well plates in culture medium with 15% FBS containing 0.3% agar layered on top of 0.6% agar. The plates were incubated at 37°C for 15 days. Colonies containing at least 50 cells were scored.

Cell migration and invasion assay

Cell migration and invasion assay were performed in Transwell chambers (24-well format, 8.0 um pore size, Millipore, Washington, DC, USA). 5 × 104 cells in serum-free media were seeded in the uncoated upper chamber for migration assay, and 1 × 105 cells in serum-free media were cultured in Matrigel (BD Biosciences, Lake Franklin, NJ, USA)-coated chamber for the cell invasion assays. The culture medium with 10% FBS was added in the lower chamber. After several hours of incubation, cells that had moved from the upper surface of membrane to the lower side were washed twice by PBS, fixed in 4% paraformaldehyde and stained in Giemsa. The number of cells were counted in five random fields (200×) under a light microscope.

Cell cycle and cell apoptosis analyses

For cell cycle assay, cells treated for 48 hours were collected, washed twice with ice-cold phosphate-buffered saline (PBS), fixed with 70% ethanol overnight, pretreated with ribonucleasea for 30minutes at room temperature and then stained with propidium iodide (PI). For cell apoptosis assay, cells treated for 48 hours were harvested, washed twice by PBS, and stained with FITC-AnnexinV and PI. Finally, Cell cycle profile and cell apoptosis profile were examined by flow cytometry (BD FACS Calibur, Becton Dickinson, San Jose, CA, USA).

Immunohistochemical assay

4 μm paraffin-embedded tissue sections were deparaffinized in xylene, dehydrated in ethanol and blocked with 3% H2O2 for 10 min. Antigen retrieval was carried out in EDTA buffer at 95°C for 15-20minutes. And sections were incubated with Ki67 antibody (Santa Cruz Inc., USA) at 4°C overnight. The secondary antibody conjugated to HRP (Santa Cruz Inc., USA) was incubated for 50 min at 37°C after the slide was washed 3 times by PBS. Finally, DAB Chromogen (Beyotime Inc., China) was added, followed by washing with water and staining in hematoxylin.

Western blot

Total proteins from tissues or cells were lysed with RIPA lysis buffer (Beyotime Inc., China), containing with phenylmethanesulfonyl fluoride (Beyotime Inc., China) and protease inhibitor cocktail (Beyotime Inc., China). 20 μg protein was separated by SDS-PAGE and transferred to a PVDF membrane (Millipore Inc., USA). Membranes were incubated with primary antibody at 4 °C overnight followed by an HRP-conjugated secondary antibody (Santa Cruz Inc., China). Anti-GAPDH antibody (Santa Cruz Inc., China) was used as an internal control. The blots were measured with ECL chromogenic substrate (Beyotime Inc., China). The following primary antibodies (Santa Cruz Inc., USA) were used: anti-N-cadherin, DSP, Vimentin, β-Catenin, c-Myc, E-cadherin, MMP-9, VEGF, α-SMA, β-catenin and Lamin B1.
