# Supplementary material for: LncRNA MIR4435-2HG targets desmoplakin and promotes growth and metastasis of gastric cancer by activating Wnt/β-catenin signaling
Source: Aging (Albany NY). 2019 Sep 4;11(17):6657–73. doi: 10.18632/aging.102164 (PMC6756883; doi:10.18632/aging.102164)
Supplement: Supplementary Figures [file aging-11-102164-s003.pdf]

SUPPLEMENTARY FIGURES

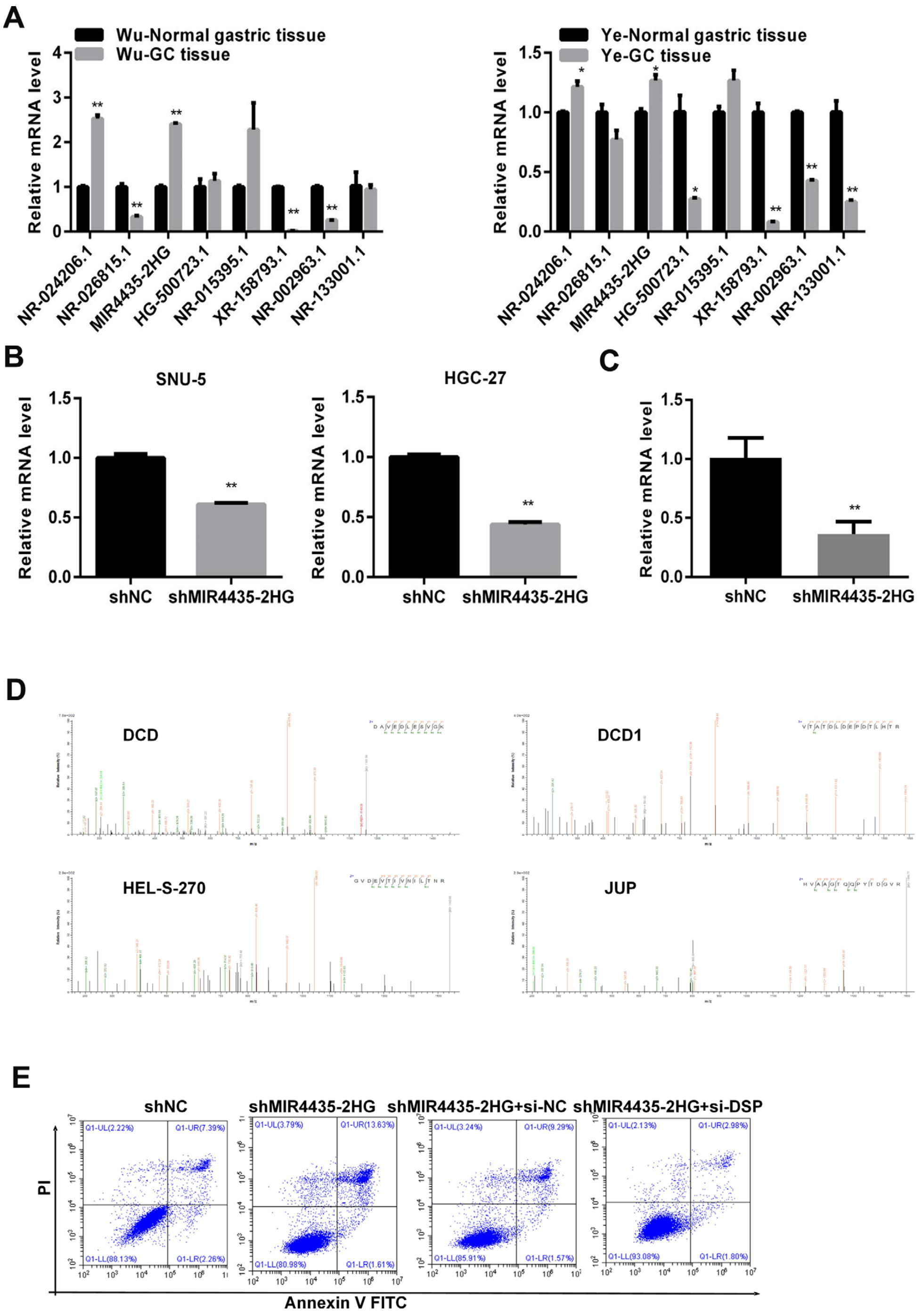

Supplementary Figure 1. (**A**) Validation of the differential expression of 8 lncRNAs in Wu and Ye cancer tissues and paired normal gastric tissues using RT-qPCR (*P < 0.05, **P < 0.01) respectively. (**B**) SNU5 and HGC-27 cells were transfection with MIR4435-2HG-specific shRNA (shMIR4435-2HG), respectively. The level of MIR4435-2HG in SNU5 or HGC-27 cells were detected with qRT-PCR. (**C**) HGC-27 cells stably transfected with sh-MIR4435 were inoculated into nude mice. The level of MIR4435-2HG in tumor tissues was detected with qRT-PCR *in vivo*. (**D**) The biotinylated sense MIR4435-2HG band was excised, digested, and subjected to mass spectrometry, which identified DCD, DCD1, HEL-S-270, and JUP as the MIR4435-2HG-interacting proteins. (**E**) The incidence of apoptosis among HGC-27 cells co-transfected with shMIR44352HG and si-DSP was determined using flow cytometry (**P < 0.01).

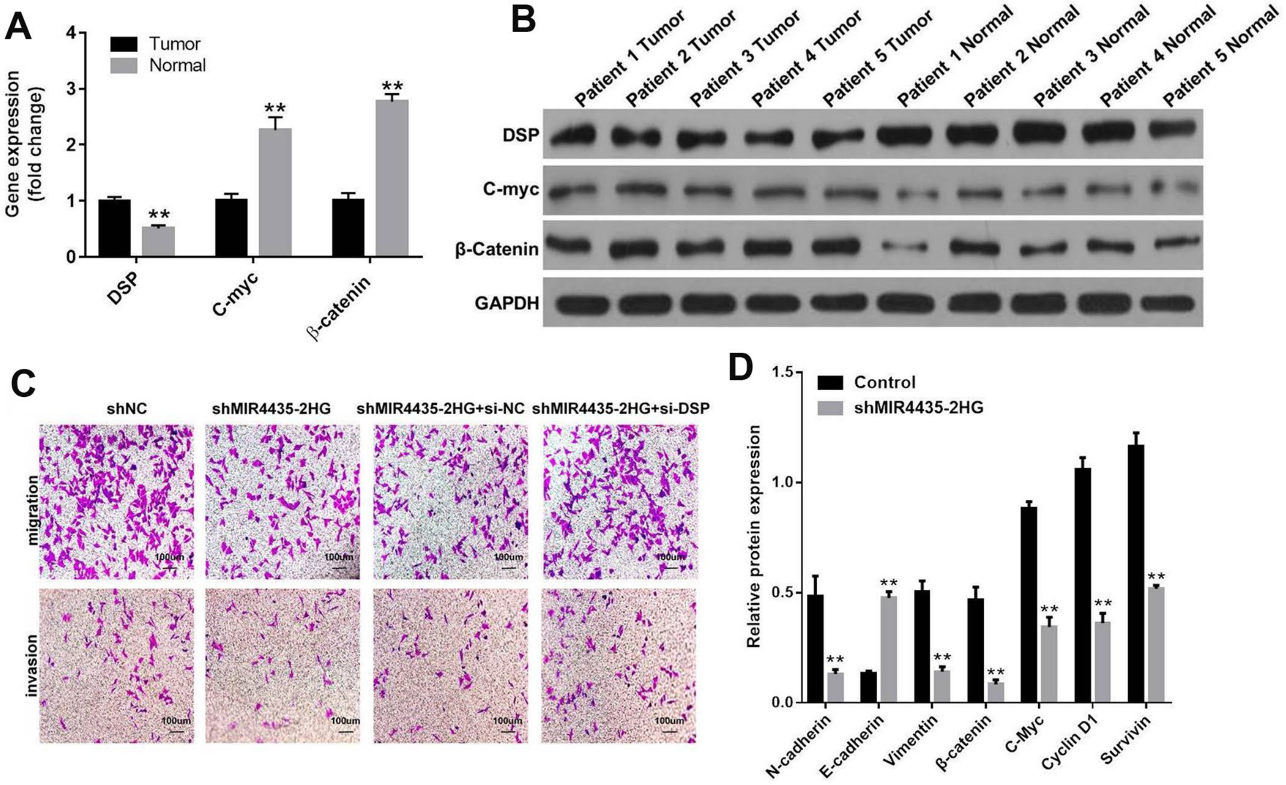

Supplementary Figure 2. (**A**, **B**) The expressions of DSP, c-myc and β-catenin in 5 GC samples and 5 adjacent normal gastric samples were detected using with qRT-PCR and WB. (**B**) Transwell assays were used to assess cell migration and invasion in the group co-transfected with shMIR4435-2HG and si-DSP (*$**P < 0.01$*). The migratory and invasive cells were observed with a light microscope. (**C**) Levels of N-cadherin, E-cadherin, vimentin, β-catenin, c-Myc, cyclin D1 and survivin within tumor tissue were determined using western blot. The relative expressions of N-cadherin, E-cadherin, vimentin, β-catenin, c-Myc, cyclin D1 and survivin were quantified via normalization to GAPDH. $**P < 0.01$ vs. shNC group.
