# Supplementary material for: LncRNA MIR4435-2HG targets desmoplakin and promotes growth and metastasis of gastric cancer by activating Wnt/β-catenin signaling
Source: Aging (Albany NY). 2019 Sep 4;11(17):6657–73. doi: 10.18632/aging.102164 (PMC6756883; doi:10.18632/aging.102164)
Supplement: Supplementary Tables [file aging-11-102164-s001.pdf]

SUPPLEMENTARY TABLES

Supplementary Table 1. Primer sequences used for and RT-qPCR analyses.

	Forward primer (5′-3′)	Reverse primer (5′-3′)
NR_024206.1	GACATTCCAGACAAGCGGTG	CCAGGGAATCTTTCAGCTGC
NR_026815.1	ACTTGAAGCCCGGTAAACCC	TCTGCTCCTGGAATATGGCC
NR_024373.1	AGACAAGCGGTGCCTGAG	ATCTTTCAGCTGCATTCCGG
HG_500723.1	ACTCCATCTCCAGTGCTTCC	GGCTACCTTGAGAACCTGGT
NR_015395.1	AAGCAGACACTATCCCAGCC	GGGTGGTGGCAAATTAGTGG
XR_158793.1	ACTCCTGTAACCCCATTTGG	AGGAGGCAAGACAGTGGATC
NR_002963.1	CTCCATGTATCTTTGGGACCTG	GGTGACAGCTTTGCCAATAAAC
NR_133001.1	TGGGTCTTGCTTACGTGGAT	TGACCCTGGTGCCTCTAAAG
DCD	TTAACAGGTGGCTCTGAGGTGGGAGA	GCTTCTGTGTGCTGGAGTGGGTATGC
DSC1	CAATCTGATGCTGCACAGAATTACAC	GCAAACTGTTCATATTTCTCACGGTC
DSP	CCAACTTGTCCTCAATCAGCATCCAG	GCTTCAGTAGACTGCGCCTCTTCAAA
HEL-S-270	GCAAAGGGTAGAAGAGCAGAGGATGG	ATCAAATACTTTCTGGAGGTGGGGCA
JUP	GCGTACCATGCAGAATACCAGCGACC	AGAACAGGACCGACTCCACAGGGGAG
HRNR	TCATTCAAGTTGGAGTGCAGGAGAGA	GAAGACTGATGGGAGTCGGAGTTTTG
GAPDH	GGAGCGAGATCCCTCCAAAAT	GGCTGTTGTCATACTTCTCATGG

Supplementary Table 2. Sequence used for transduction and RNA-pull-down.

	Sequence (5′-3′)
MIR4435-2HG shRNA	gatcggtctggtcggtttcccattttcaagagaaaatgggaaaccgaccagaccttttttaatt
DSP siRNA	Sense: gacaugaaucaguaaguaatt<br>Anti-sense: uuacuuacugauucauguctt
Pulldown MIR4435-2HG sense transcription	Sense: taatacgactcactatagggagcatgagtcatctcgttcc<br>Anti-sense: tttttttttttttttctgttttctttagttttgcttttaatg
Pulldown MIR4435-2HG anti-sense transcription	Sense: taatacgactcactatagggtttttttttttttttctgttttctttagttttgcttttaatg<br>Anti-sense: agcatgagtcatctcgttccaatg
